# Prevalence and Risk Factors of Sarcopenia among Older Adults Aged ≥65 Years Admitted to Daycare Centers of Taiwan: Using AWGS 2019 Guidelines

**DOI:** 10.3390/ijerph18168299

**Published:** 2021-08-05

**Authors:** Cheng-Fen Chang, Yu-Lyu Yeh, Huang-Yu Chang, Sheng-Hua Tsai, Jiun-Yi Wang

**Affiliations:** 1 Department of Healthcare Administration, Asia University, Taichung 41354, Taiwan; cfchang222@gmail.com (C.-F.C.); yyyeh@asia.edu.tw (Y.-L.Y.); 2 Department of Nursing, Ching Kuo Institute of Management and Health, Keelung 203301, Taiwan; 3 Department of Dietetics, Keelung Hospital, Ministry of Health and Welfare, Keelung 20148, Taiwan; kln00852@kln.mohw.gov.tw; 4 Department of Social Work, Keelung Hospital, Ministry of Health and Welfare, Keelung 20148, Taiwan; ysocialwork@gmail.com; 5 Department of Medical Research, China Medical University Hospital, China Medical University, Taichung 404332, Taiwan

**Keywords:** sarcopenia, prevalence, older adults, standing balance, daycare center

## Abstract

Sarcopenia is a geriatric syndrome which is likely to cause disability, body unbalance, and mortality and thus can lead to heavy healthcare expenditure and caregiver burden. Although some studies have addressed the prevalence of sarcopenia for older adults, there are limited studies conducted in daycare centers. The present study aimed to (i) estimate the prevalence of sarcopenia and (ii) explore associated factors of sarcopenia and standing balance among older adults admitted to daycare centers in Taiwan. The cross-sectional study collected data on demographics, health status, handgrip strength, gait speed (GS), skeletal muscle mass, Taiwan-Mini Nutritional Assessment Short-Form (TW-MNA-SF), and Short Physical Performance Battery from daycare centers in northern Taiwan. The definition of sarcopenia followed the Asian Working Group for Sarcopenia 2019 guidelines. Among 173 participants ≥65 year-old, 50.9% had confirmed sarcopenia, 47.4% possible sarcopenia, and 1.7% normal. Results showed that calf circumference, TW-MNA-SF, dementia, and body mass index (BMI) were associated with sarcopenia. Moreover, BMI, GS, and sarcopenia were associated factors of standing balance. The study estimated a high prevalence of sarcopenia in daycare centers and identified some significant factors of sarcopenia and standing balance. Early nutritional and physiotherapy interventions could benefit older adults to prevent sarcopenia or unbalance.

## 1. Introduction

In recent years, sarcopenia in older adults has become an important healthcare issue worldwide. Sarcopenia is a geriatric syndrome that could be caused by aging, body composition change and chronic diseases [1]. A high prevalence of sarcopenia can cause heavy healthcare expenditure and caregiver burden [2,3]. Many studies have indicated that older adults with sarcopenia were associated with increased falling rate, comorbidities, malnutrition, functional disabilities and mortality rate [4,5].

In Europe and the USA, the prevalence of sarcopenia with different diagnostic criteria ranged from 4.6% to 43% in communities [6,7] and from 23% to 68% in clinical settings [8,9,10,11,12]. In Taiwan, the prevalence of sarcopenia was recently reported as ranging from 6.7% to 10% in communities [3,13,14] and reaching 50% in clinical settings [15]. The wide ranges of prevalence were not only due to different settings and populations, but also the variety of diagnostic criteria being used.

Consensus on the definition criteria of sarcopenia was not available until 2010. The European Working Group on Sarcopenia in Older People (EWGSOP) reached a consensus and started to unify the criteria for sarcopenia. In 2014, the Asian Working Group for Sarcopenia (AWGS) developed compatible evaluation criteria for Asian populations based on the characteristics of different ethnicities, body size, lifestyles, and cultural backgrounds [16]. Later, in response to additional research and assessment of community need, AWGS adjusted their diagnostic guideline and developed a new one in 2019 [17]. According to the newest guidelines, confirmed sarcopenia is defined as low muscle mass, plus low muscle strength or low physical performance, and possible sarcopenia is defined as either low muscle strength or low physical performance.

To cope with the rapid growth of an aging population, the Taiwanese government has developed several health policies for older adults. One of these is the establishment of daycare centers which aim to help older adults to age in situ. The services of daycare centers include ADL-assisted care, ADL-training, health promotion, leisure activities, transportation services, rehabilitation services, fitness-training and nutritional meal services. Criteria for enrolling to daycare centers include older adults with disability, frailty, physical and mental impairment, and dementia [18]. Because of the potential difference in health conditions between older adults from the general community and daycare centers, studies of sarcopenia in daycare centers appear significant and relevant.

In addition to prevalence, the risk factors of sarcopenia are also worth exploring. It is well known that sarcopenia is often accompanied by less muscle strength, lower limb function, frailty, gait instability, lower calf circumference (c.c.), and lower body mass index (BMI) [19]. Protein synthesis, nutritional unbalance, endocrine function, loss of motor neurons, and hormonal status may have contributed to pathogenesis [20,21]. Decreased physical function or physical performance has been identified as a risk factor [22]. Some diseases, such as dementia, osteoporosis, cardiovascular-related diseases, depression, obesity, and calorie or protein deficiency could also be associated with sarcopenia [23,24,25].

Despite the fact that many studies have addressed the prevalence and risk factors of sarcopenia for older adults in communities and clinical settings, there are limited studies conducted in daycare centers, especially in Taiwan. Therefore, the aims of the present study were to (i) estimate the prevalence of sarcopenia and (ii) explore associated factors of sarcopenia and standing balance among older adults admitted to daycare centers in Taiwan.

## 2. Materials and Methods

### 2.1. Study Design and Recruitment

The study was approved by the Ethics Committee of the Ministry of Health and Welfare at Taipei Hospital (registration ID No.TH-IRB-0019-0046). Written informed consent was obtained from all participants before enrollment in this study. This study was a cross-sectional research study conducted at daycare centers in Keelung City, a city in northern Taiwan with 8 daycare centers. Data were collected between August and September 2020 from all the 8 daycare centers in the city. Participants completed written questionnaires and assessments for confirmed sarcopenia during the study period.

**Figure 1:**
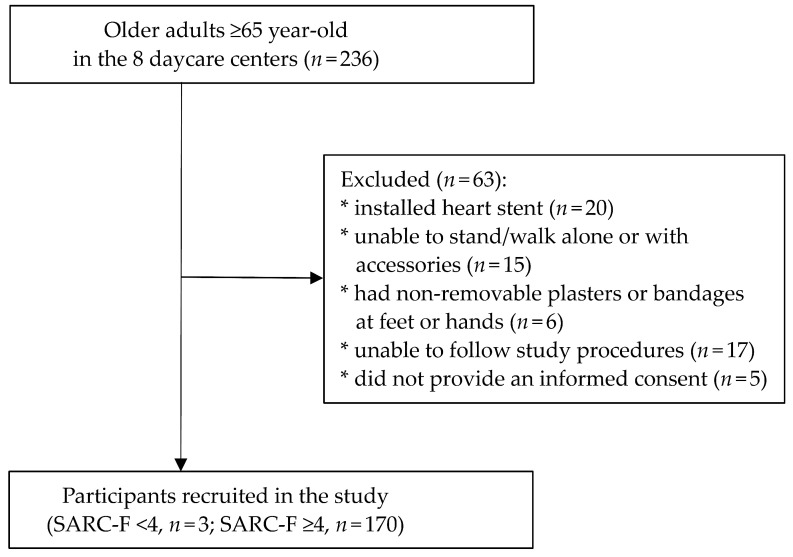
Flow chart of the selection of study participants.

The inclusion criteria for the study were: (i) aged ≥65 years, (ii) able to communicate in Mandarin Chinese or Taiwanese, (iii) willing to provide an informed consent, or if unable, a proxy-informed consent obtained from their substitute decision maker. The exclusion criteria were: (i) installed heart stent in the body, (ii) unable to stand/walk alone or with accessories, (iii) having non-removable plasters or bandages at feet or hands and (iv) unable to follow study procedures (e.g., due to delirium or language barriers). There were 236 older adults ≥65 year-old reachable from the 8 daycare centers. Among them, 63 were ineligible and were excluded. Three of the remaining 173 participants had SRAC-F <4, thus they were identified as non-sarcopenia and were not further assessed. Consequently, data of 173 participants were used to calculate the prevalence of sarcopenia, and 170 participants were included in the other analyses. Figure 1 shows the flow chart of the selection procedure.

### 2.2. Assessment of Sarcopenia

The AWGS 2019 consensus defined sarcopenia as low muscle mass, with low muscle strength, or low physical performance [17]. There are two levels of sarcopenia, possible sarcopenia and confirmed sarcopenia. Possible sarcopenia was defined as low muscle strength or low physical performance and confirmed sarcopenia was defined as low muscle mass plus low muscle strength or low physical performance. When low muscle mass, low muscle strength, and low physical performance occurs simultaneously, it is further defined as severe sarcopenia. As suggested in the AWGS 2019 guideline, a higher score of SARC-F or SARC-CalF or lower calf circumference should be used for case finding in health care or community services settings [17]. In the present study, participants with SARC-F ≥4 would be further assessed for sarcopenia.

Muscle mass was assessed by measuring skeletal muscle mass index (SMI) which was calculated by appendicular skeletal muscle mass (ASM) divided by height (m) squared. ASM was calculated by the sum of muscle mass in the arms and legs using bioelectrical impedance analysis (BIA) (InBody 230; Biospace Co. Ltd., Seoul, Korea). Low muscle mass was defined with cut-off points of <7.0 kg/m^2^ for male and <5.7 kg/m^2^ for female. Muscle strength was assessed by measuring the power of handgrip strength (HGS) using the Camry pneumatic hand dynamometer (EH101; Xiangshan Inc., Guangdong, China). Each hand pressed hold on the hand dynamometer for 3–5 s and the result from the strongest hand was used for analyses. Low HGS was defined with cut-off points of <28 kg for male and <18 kg for female. Six-meter gait speed (GS) <1 m/s was used to define low physical performance in the present study.

### 2.3. Assessment of Short Physical Performance Battery (SPPB) and Standing Balance

The SPPB is a critical, efficient, practical, and safe assessment for physical functioning and disability for older adults [26]. The SPPB is a composite measure assessing sit-to-stand, GS, and standing balance performance. It was calculated from three components: time to rise from a chair 5 times; time to complete a 4 m GS, and the ability to stand for up to 10 s with feet positioned in three different ways (together side-by-side, semi-tandem and tandem) [27]. The 5 times sit to stand test score was defined as: <11.19 s, score = 4; 11.20–13.69 s, score = 3; 13.70–16.69 s, score = 2; 16.7–60 s, score = 1; tried but unable, score = 0). The 4 m GS was defined as: <4.82 s, score = 4; 4.82–6.20 s, score = 3; 6.21–8.7 s, score = 2; >8.7 s, score = 1; tried but unable, score = 0). The standing balance score was calculated by adding up three subtests which included full tandem (0–2 s, score = 0; 3–9 s, score = 1; 10 s, score = 2), semi-tandem (<10 s, score = 0; >10 s, score = 1), and side-by-side stands (<10 s, score = 0; >10 s, score = 1) [27,28]. The SPPB score was calculated by summing the three individual categorical scores (range: 0–12), with higher scores indicating better body function.

### 2.4. Assessment of Other Covariates Score

Basic demographic characteristics, anthropometric measures, duration of participation in daycare centers, comorbidities, and nutrition status were collected from the participants. Data on comorbidities, including hypertension, coronary heart disease, dementia, stroke, and osteoporosis, were retrieved according to participants’ medical records. The Taiwan-Mini Nutritional Assessment Short Form (TW-MNA-SF) [29] was used as a screening tool to evaluate nutritional status. Height (cm), c.c.(cm) was collected using standard methods. Body weight (kg) and BMI (kg/cm^2^) were measured by BIA. The other variables were coded into the following categories for analysis purpose, respectively: age: 65–74 years (reference), 75–84 years and ≥85 years; BMI:  <21, 21–24 (as reference), >24; c.c.: normal, low c.c. (<34 cm for males and <33 cm for females); TW-MNA-SF: normal (score > 11) and at risk of malnutrition (score ≤ 11) [17].

### 2.5. Statistical Analysis

All statistical analyses were performed using IBM SPSS Statistics v.27. Descriptive statistics were used to show distributions of demographic characteristics and other studied variables. The χ^2^ test was used to compare the prevalence of sarcopenia between males and females. A binary logistic regression model with the inclusion of gender, age, BMI, lower c.c., risk of malnutrition, lower SPPB score, and dementia was conducted to explore significantly associated factors of confirmed sarcopenia. A multiple regression model was performed to assess the correlations between standing balance and demographic characteristics, risk of malnutrition, 5-times sit-to-stand test, 4 m GS, and sarcopenia. The significance level was set at 0.05 for all testing hypotheses.

## 3. Results

### 3.1. Demographic Characteristics

Participants’ general characteristics, comorbidities, specific diseases and geriatric assessment are presented in Table 1. The mean age of participants was 81.6 ± 6.8 years, with females comprising 75% of total participants. Over half of participants were low c.c. Average duration of participation in daycare centers was 24.1 ± 17.8 month. On average, participants had 3.0 ± 1.7 kinds of comorbidity. More than half of the participants had dementia and hypertension. Majority of the participants (78.8%) have normal nutrition intake based on the TW-MNA-SF score. Almost all of the participants (95.9%) have low body function based on the SPPB score. Although males had higher HGS and SMI score compared to females, the difference in the sarcopenia definition criteria (Table 2) still put males at higher risk of getting sarcopenia. The scores of participants’ HGS, GS, and SMI were 21.1 ± 6.4 kg, 0.40 ± 0.20 m/s and 7.0 ± 1.7 kg/m^2^ for male; 12.7 ± 5.0 kg, 0.40 ± 0.20 m/s and 5.9 ± 1.3 kg/m^2^ for female, respectively.

### 3.2. Prevalence of Possible and Confirmed Sarcopenia

Out of the 173 participants, 82 participants were classified as possible sarcopenia (47.4%; 95% CI 40.1 to 54.8), 88 participants were classified as confirmed sarcopenia (50.9%; 95% CI 43.5 to 58.2), and three participants were classified as normal (1.7%; 95% CI 0.6 to 5.0) according to the criteria of AWGS 2019. Gender difference in prevalence and definition criteria of sarcopenia are presented in Table 2. The majority of participants had low muscle strength (77.5%) and low physical performance (97.1%). Half of the participants (50.9%) had low muscle mass. Compared to females, males showed a lower prevalence of possible sarcopenia (32.6% and 52.3%, respectively, *p* = 0.025) and a higher prevalence of confirmed sarcopenia (65.1% and 46.2%, respectively, *p* = 0.031).

### 3.3. Associated Parameters of Sarcopenia

To identify effect relationship between sarcopenia and low SPPB, gender, age, BMI, low c.c., at risk of malnutrition and dementia, logistic regression analysis was performed. The purpose of the analysis was to explore the relationship of the risk factors and sarcopenia. Low SPPB score (≤9) (OR = 1.74; 95% CI 0.22 to 13.87, *p* = 0.602) was not significantly different between confirmed sarcopenia and possible sarcopenia. Participants with higher BMI were less likely to have confirmed sarcopenia (OR = 0.22; 95% CI 0.09 to 0.55, *p* = 0.001). Males (OR = 3.09; 95% CI 1.19 to 7.98, *p* = 0.020), those with low c.c. (OR = 3.39; 95% CI 1.49 to 7.71, *p* = 0.004), at risk of malnutrition (OR = 5.65; 95% CI 1.96 to 16.31, *p* = 0.001) and dementia (OR = 2.31; 95% CI 1.05 to 5.04, *p* = 0.036) were more likely to have confirmed sarcopenia (Table 3).

### 3.4. Associated Parameters of Standing Balance

Table 4 shows the result of the multiple regression model for standing balance and risk factors of sarcopenia. Among all factors, higher BMI, 4 m GS and sarcopenia were significantly associated with standing balance. Participants with higher BMI (B = −0.78; 95% CI −1.26 to −0.30, *p* = 0.002) were more unbalanced than participants with normal BMI. In addition, participants with low 4 m GS (B = −0.03; 95% CI −0.06 to −0.01, *p* = 0.011) were more balanced. Participants with sarcopenia (B = −0.98; 95% CI −1.42 to −0.53, *p* < 0.001) were more unbalanced.

## 4. Discussion

### 4.1. Prevalence of Possible and Confirmed Sarcopenia

By including all the daycare centers in Keelung City in the present study, the prevalence of confirmed sarcopenia for older adults admitted to daycare centers was estimated at 50.9% which is much higher than those in the community [13]. Besides those with confirmed sarcopenia, almost all of the remaining participants were considered as possible sarcopenia, which implies a high risk of sarcopenia.

Owing to the enrollment criteria, older adults admitted to daycare centers are more likely to be frail, disabled, and unhealthy than those in the general community. However, among the eight daycare centers, the prevalence of confirmed sarcopenia ranged from 27.3% to 80.0%. The wide range of prevalence implies that the prevalence in a specific daycare center varies widely. Participant characteristics or risk factors of sarcopenia deserve more attention. Nonetheless, the small number (ranging from 16–29) in each center restricts further inference. In summary, the overall estimated prevalence in daycare centers was much higher than in the community and relatively close to that in clinical settings. A recent study in Switzerland estimated a prevalence of 22.6% among hospitalized geriatric patients [9]; another study in Australia showed a prevalence of 40.2% among residents in 11 nursing homes [11]. In Taiwan, the prevalence of sarcopenia among older adults in the community-dwelling was estimated at 6.8% (*n* = 731, mean age 73.4  ±  5.4 years) [30], while the prevalence of sarcopenia among inpatients with hip fracture in a hospital was 50.36% (*n* = 139, mean age 80.7 years) [15].

Along with the Taiwanese government policy and promotion, establishing daycare centers for older adults in the community has become a new trend [18]. Regular screening for sarcopenia in daycare centers can provide better understanding of older adults’ health conditions and may thus help to prevent sarcopenia through proper interventions. However, screening for sarcopenia was not a requirement for daycare centers. In addition, another barrier was lack of proper tools for measuring muscle mass [18]. Based on the AWGS 2019 guideline, assessors can choose an easy to use and conventional method to assess sarcopenia. Thus, the AWGS 2019 guideline is recommended to be used in daycare centers.

### 4.2. Associated Parameters of Confirmed Sarcopenia

SPPB might not be a good instrument to identify confirmed and possible sarcopenia in daycare centers. The present study aimed to use SPPB to differentiate confirmed sarcopenia and possible sarcopenia, but found that SPPB scores (≤9) were not significantly different between confirmed and possible sarcopenia, which was not consistent with previous studies. SPPB is a quick, easily administrable, and objective measure of muscle strength and physical performance (such as gait speed and standing balance). Not all facilities have the equipment to test muscle mass and identity sarcopenia. Previous study reported using SPPB as a screening tool to diagnose those at risk of sarcopenia [31]. SPPB might be a good indicator to distinguish between sarcopenia and normal, but not between confirmed and possible sarcopenia [31]. In addition, the small sample size in the present study and ethnicity differences might also affect this result.

Gender was a significant factor related to confirmed sarcopenia. Male participants had higher likelihood of confirmed sarcopenia compared to females. Similar results have been reported previously [2,32]. Some studies mentioned that age-related decrease in sex hormones is a major contributor to loss of lean mass and increase in fat mass for both genders [33]. However, fat converted androgens to estrogens and anabolic effects only in women, thereby attenuating the loss of lean mass and strength in women, but not in men [34]. Results of the present study indicated that males had higher HGS and SMI scores than females. However, males had higher percentage of low muscle mass compared to female, based on the sarcopenia index in Table 2. Therefore, males had a higher risk of sarcopenia.

Malnutrition and lower BMI were prone to sarcopenia, consistent with previous study [9]. Malnutrition was related to low muscle mass, low muscle strength, and low physical performance, with a higher risk of developing sarcopenia [35]. In addition, lower c.c., another variable closely related to sarcopenia, also related to decreased SMI and a greater extent of malnutrition [36]. The result is consistent with a recent study which suggested that possible sarcopenia, using calf circumference for case-finding, is a strong predictor for sarcopenia [37]. To increase protein consumption to 1.2 to 1.5 g/kg/day can prevent “muscle deficiency” [8] and may prevent sarcopenia in the long term. Moreover, higher BMI indicates better nutrition intake and higher SMI (such as larger c.c.) [9]. Therefore, higher BMI was negatively associated with the prevalence of confirmed sarcopenia. However, excess visceral fat could lead to obesity sarcopenia [38,39]. Obese individuals were less physically active which resulted in a gradual decrease in muscle mass and strength [16,40]. Therefore, supplying a protein sufficient and healthy diet for older adults in daycare centers is needed to prevent sarcopenia.

Very few studies have investigated the relationship between dementia and sarcopenia. A few studies found that sarcopenia was accompanied with dementia [24,41,42] which is consistent with the present study. The present study found that around half of the older adults were diagnosed with both dementia and sarcopenia. Pathological mechanisms of dementia included reduced neuronal volume in the brain, which might cause decreased skeletal muscles mass (definition criteria for sarcopenia), and decreased cognitive functions and brain structures (such as lower IQ, smaller brain volume) [43,44,45]. Therefore, sarcopenia might be a result of cognitive decline [44]. The treatment and prophylaxis for cognitive decline might also prevent the development of sarcopenia.

### 4.3. Associated Parameters of Standing Balance

In the present study, there was a negative association between standing balance and higher BMI, slower GS, and sarcopenia, similar to other studies [46,47]. Higher BMI caused by excess body fat could exacerbate fat infiltration into muscle and lead to gradual decrease of muscle quality, physical performance and standing balance [16,40]. Low physical performance is a risk factor for sarcopenia and functional deterioration, including declines in gait and balance, increased fall risks, and loss of independence [48]. Therefore, maintaining a healthy weight is important for older adults, which could avoid sarcopenia and further maintain body balance.

The pathogenesis of balance might involve less active neuromuscular junctions, especially with loss of type II muscle fiber. In addition, the vestibular system and cerebellum are thought to play primary roles in postural control. The cerebellum is important for modifying limb and trunk movements and balancing muscle strengths for a required task. Then, situational cues and prior experiences modify these inputs and contribute to balance control [20,49].

Impaired standing balance and slower GS were associated with increased incidence of falls in older adults. Furthermore, falls were associated with significantly higher morbidity and mortality in older adults [50]. Falls caused by impaired balance was the most common cause of accidental death and nonfatal accidental injury in those over 65 years old, accounting for the second highest number of accidental deaths [51]. Figueiro et al. [52] found that older adults at high risk of falling demonstrated a significantly slower GS than adults at low risk of falling. Wolfson et al. Ref. [53] also indicated that decreased muscle strength could damage balance in older adults. When muscle strength increased by each newton meter per kg, the odds of losing balance decreased by 20%. The negative association between standing balance and lower muscle strength or sarcopenia is consistent with the present study.

Finally, higher BMI, slower GS, and sarcopenia might be risk factors for impaired standing balance. Disability, falling and reduced quality of life accompany standing balance decline. Therefore, older adults are strongly encouraged to maintain ideal body weight and good nutritional balance, especially with an adequate amount of protein intake to increase limb muscle and maintain body balance.

### 4.4. Strengths and Limitations

To the author’s best knowledge, this is the first study in Taiwan to investigate the prevalence and risk factors of sarcopenia in daycare centers based on the AWGS 2019 guideline. The findings provide better understanding of the sarcopenia status of older adults in daycare centers. This could influence health policy makers and managers of daycare centers to pay more attention to relevant issues regarding sarcopenia. In addition, this study sampled all daycare centers in Keelung City. Thus the results should represent older adults admitted to daycare centers in the city.

There are several limitations in our study. First, causation of the risk factors of possible sarcopenia and confirmed sarcopenia could not be explained due to the nature of the cross-sectional study design. Future research studying the causes of confirmed sarcopenia is warranted. Second, our data lack generalization. The present study participants were only from one city of Taiwan and cannot represent the entire population. Future study that includes more cities is needed. In addition, expanding the study design into a longitudinal research study could discover the order of influence of the relevant factors. Third, the present study used BIA to measure muscle mass. Some research has indicated that using Dual-energy X-ray absorptiometry to measure muscle is more accurate, as BIA could overestimate muscle mass and lead to underestimate of sarcopenia [54]. However, BIA can be easily operated, is portable and inexpensive to use compared to Dual-energy X-ray absorptiometry, and is also widely used in both community and clinical settings.

## 5. Conclusions

A high prevalence of confirmed sarcopenia (50.9%) in daycare centers in northern Taiwan was observed using the AWGS 2019 guideline. Gender, higher BMI, lower calf circumference and lower nutritional score were significant risk factors for confirmed sarcopenia. Higher BMI, GS and sarcopenia were significantly associated with standing balance. More attention should be paid to muscle mass, muscle strength, and physical performance of older adults in daycare centers. Moreover, lifestyle intervention may be beneficial for such a population to decrease the risk of confirmed sarcopenia and delay functional disability.

## Figures and Tables

**Table 1:** Baseline characteristics of the study population, stratified by gender (*n* = 170).

Characteristic	All N = 170	Male N = 42 (24.7%)	Female N = 128 (75.3%)	*p*-Value
General characteristic				
Age (years)				
65–74	26 (15.3)	10 (23.8)	16 (12.5)	0.005
75–84	77 (45.3)	10 (23.8)	67 (52.3)	
≥85	67 (39.4)	22 (52.4)	45 (35.2)	
M ± SD	81.6 ± 6.8	82 ± 7.7	81.4 ± 6.6	0.641
BMI (kg/m^2^)	48 (28.2) 55 (32.4) 67 (39.4) 23.3 ± 3.7	12 (28.6) 13 (31.0) 17 (40.5) 23.0 ± 3.0	36 (28.1) 42 (32.8) 50 (39.1) 23.4 ± 4.0	
<21				0.974
21–24				
≥24				
M ± SD				0.564
Calf circumference (cm)	89 (52.4) 81 (47.6) 32.3 ± 3.2	22 (52.4) 20 (47.6) 33.2 ± 3.6	67 (52.3) 61 (47.7) 32.0 ± 3.0	
<34 (33) for males (females)				0.997
≥34 (33) for males (females)				
M ± SD				0.036
Duration of participation in daycare centers (m), M ± SD	24.1 ± 17.8	21.2 ± 16.4	25.0 ± 18.2	0.228
Comorbidities (n), M ± SD	3.0 ± 1.7	3.2 ± 1.7	2.9 ± 1.7	0.303
Hypertension	89 (52.4)	22 (52.4)	67 (53.2)	0.997
Coronary heart disease	50 (29.4)	17 (40.5)	33 (25.8)	0.070 0.109 0.007 0.237
Dementia	95 (55.9)	19 (45.2)	76 (59.4)	
Stroke	15 (8.9)	8 (19.1)	7 (5.5)	
Osteoporosis	21 (12.5)	3 (7.1)	18 (14.1)	
Geriatric assessment parameters				
TW-MNA-SF(score)				
Normal (>11)	134 (78.8)	30 (71.4)	104 (81.3)	0.176
At risk and malnutrition (≤11)	36 (21.2)	12 (28.6)	24 (18.8)	
M ± SD	12.5 ± 1.7	12.3 ± 1.6	12.6 ± 1.8	0.282
SPPB				
Low SPPB score (≤9)	163 (95.9)	41 (97.6)	122 (95.3)	0.514
Normal (>9)	7 (4.1)	1 (2.4)	6 (4.7)	
M ± SD	5.7 ± 2.2	5.4 ± 2.5	5.9± 2.1	0.300
Balance (score), M ± SD	2.6 ± 1.4	2.4 ± 1.6	2.7 ± 1.3	0.260
4 m GS, M ± SD	13.0 ± 8.1	13.9 ± 10.2	12.7 ± 7.4	0.440
5-times sit to stand test (score)				
<12	5 (2.9)	1 (2.4)	4 (3.1)	0.804
≥12	165 (97.1)	41 (97.6)	124 (96.9)	
M ± SD	21.3± 11.3	23.8 ± 13.2	20.5 ± 10.5	0.104
Sarcopenia index	14.8 ± 6.5 0.4 ± 0.2 6.2 ±1.5	21.1 ± 6.4 0.4 ± 0.2 7.0 ± 1.7	12.7 ± 5.0 0.4 ± 0.2 5.9 ± 1.3	
HGS (kg), M ± SD				<0.001
GS (m/s), M ± SD				0.905
SMI (kg/m^2^), M ± SD				<0.001

BMI body mass index, TW-MNA-SF Taiwan Mini Nutrition Assessment-Short Form, SPPB Short Physical Performance Battery, HGS Handgrip strength, GS gait speed, SMI skeletal muscle mass index.

**Table 2:** Prevalence of sarcopenia, stratified by gender (*n* = 173).

Prevalence of Sarcopenia	All N = 173	Male N = 43	Female N = 130	*p*-Value
Sarcopenia Definition Criteria				
Low muscle strength, *n* (%)	134 (77.5)	34 (79.1)	100 (76.9)	0.770
Low physical performance, *n* (%)	168 (97.1)	42 (97.7)	126 (96.9)	0.799
Low muscle mass, *n* (%)	88 (50.9)	28 (65.1)	60 (46.2)	0.031
Prevalence of sarcopenia				0.025 0.031 0.145
Possible sarcopenia, *n* (%)	82 (47.4)	14 (32.6)	68 (52.3)	
95% CI (%)	(40.1–54.8)	(4.9–13.1)	(32.3–46.7)	
Confirmed sarcopenia, *n* (%)	88 (50.9)	28 (65.1)	60 (46.2)	
95% CI (%)	(43.5–58.2)	(11.4–22.4)	(28.0–42.0)	
Severe sarcopenia ^1^, *n* (%)	76 (43.9)	23 (53.8)	53 (40.8)	
95% CI (%)	(36.8–51.4)	(9.0–19.2)	(24.3–37.9)	

95% CI 95% confidence interval. ^1^ Severe sarcopenia was included in confirmed sarcopenia.

**Table 3:** Associated parameters of confirmed sarcopenia among older adults in daycare centers (*n* = 170).

Parameter	*n*	OR	95% CI	*p*-Value
Male	43	3.09	1.19–7.98	0.020
Age (year)				
65–74	26	1.00 (ref)	-	-
75–84	77	1.19	0.40–3.52	0.749
≥85	67	2.46	0.81–7.43	0.112
BMI (kg/m^2^)				
<21	48	0.94	0.34–2.54	0.895
21–24	55	1.00 (ref)	-	-
≥24	67	0.22	0.09–0.55	0.001
Low c.c. ^1^	89	3.39	1.49–7.71	0.004
At risk of malnutrition ^2^	36	5.65	1.96–16.31	0.001
Low SPPB score (≤9)	163	1.74	0.22–13.87	0.602
Dementia	97	2.31	1.05–5.04	0.036

BMI body mass index, c.c. calf circumference, SPPB Short Physical Performance Battery, OR odds ratio, 95% CI 95% confidence interval. ^1^ Defined as c.c. < 34 cm and <33 cm for males and females. ^2^ Defined as TW-MNA-SF score ≤ 11.

**Table 4:** Analysis of the correlation between the parameters of sarcopenia and standing balance among older adults at daycare centers (*n* = 170).

Parameter	*n*	B	95% CI	*p*-Value
Male	43	−0.02	−0.48 to 0.44	0.932
Age (year)				
65–74	26	1.00 (ref)	-	-
75–84	77	−0.05	−0.62 to 0.52	0.856
≥85	67	−0.08	−0.67 to 0.51	0.792
BMI (kg/m^2^)				
<21	48	0.03	−0.48 to 0.54	0.909
21–24	55	1.00 (ref)	-	-
≥24	67	−0.78	−1.26 to −0.30	0.002
Low c.c.^1^	89	−0.04	−0.49 to 0.41	0.856
At risk of malnutrition^2^	36	0.03	−0.46 to 0.51	0.904
5-times sit to stand test (score)	170	−0.02	−0.03 to 0.00	0.110
4m GS	170	−0.03	−0.06 to −0.01	0.011
Sarcopenia	88	−0.98	−1.42 to −0.53	<0.001

BMI body mass index, c.c. calf circumference, GS gait speed; R^2^ = 0.188; adjusted R^2^ = 0.136. ^1^ Defined as c.c. < 34 cm and <33 cm for males and females. ^2^ Defined as TW-MNA-SF score ≤ 11.

## Data Availability

The datasets generated during and analyzed during this study are available from the corresponding author on reasonable request.
